# Same-session endoscopic ultrasound-guided gallbladder drainage for acute cholecystitis with lumen-apposing metal stent and rendezvous for biliary stones in invisible papilla

**DOI:** 10.1055/a-2587-8500

**Published:** 2025-05-14

**Authors:** Davide Scalvini, Aurelio Mauro, Stefano Mazza, Carlo Ciccioli, Marco Bardone, Francesca Torello Viera, Andrea Anderloni

**Affiliations:** 1 19001 Department of Internal Medicine and Therapeutics, University of Pavia, Pavia, Italy; 2 18631 Gastroenterology and Digestive Endoscopy Unit, Fondazione IRCCS Policlinico San Matteo, Pavia, Italy; 3 18998 Section of Gastroenterology and Hepatology, PROMISE, University of Palermo, Palermo, Italy

EUS-guided gallbladder drainage (EUS-GBD) is increasing its use for distal biliary malignant obstruction and in case of acute cholecystitis in frail or unfit for surgery patients 1 . However, in the case of concomitant choledocholithiasis, an ERCP is usually necessary to remove biliary stones, and new techniques have been explored 2 . In this video-case report, we present a single-session EUS-GB for acute cholecystitis with LAMS and an innovative biliary stones clearance with a gallbladder rendezvous ( Video 1 ).

Video of a EUS-gallbladder drainage for acute cholecystitis with lumen-apposing metal stent and a gallbladder rendezvous for biliary stones in invisible papilla done in a single session.Video 1

This case involves an 85 year-old woman who was admitted for acute cholecystitis and common bile duct (CBD) stones, after multidisciplinary evaluation, she has been considered frail and unfit for surgery, thus an EUS-GB and an ERCP were proposed.

ERCP was started but it was suspended for inability to find the major papilla which seems to be dislocated by a duodenal diverticulum ( Fig. 1 ). EUS-GBD was effectively performed from the antrum, with a 15 × 10 mm EC-LAMS (Hot Axios, Boston Scientific) ( Fig. 2 ). Subsequently, a large balloon dilation was employed to dilate the LAMS up to 15 mm and to do a direct cholecystoscopy with a standard gastroscope ( Fig. 3 ). Using a 0.035 × 450 cm guidewire on a sphincterotome, the cystic duct was cannulated, and then the guidewire was advanced anterograde into the CBD and further into the duodenum. Anterograde cholangiography confirmed the presence of choledocholithiasis. The papilla was anterograde dilated with a balloon up to 12 mm, and the guidewire was maintained in the duodenum. Then, a duodenoscope with a lateral-view was reintroduced, and, with the guidewire still in place to facilitate an easier cannulation ( Fig. 4 ), the CBD was cleared from three small stones using an extractor balloon.

**Fig. 1:**
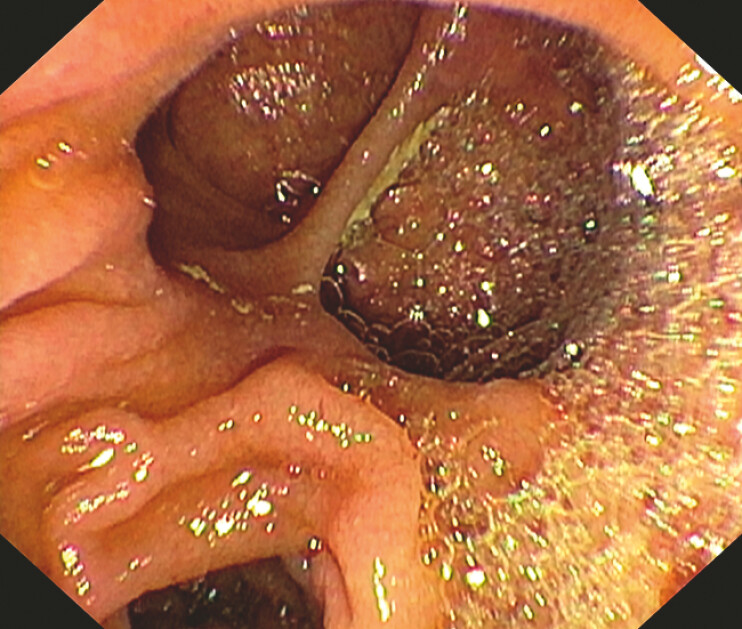
Invisible papilla dislocated by a duodenal diverticulum.

**Fig. 2:**
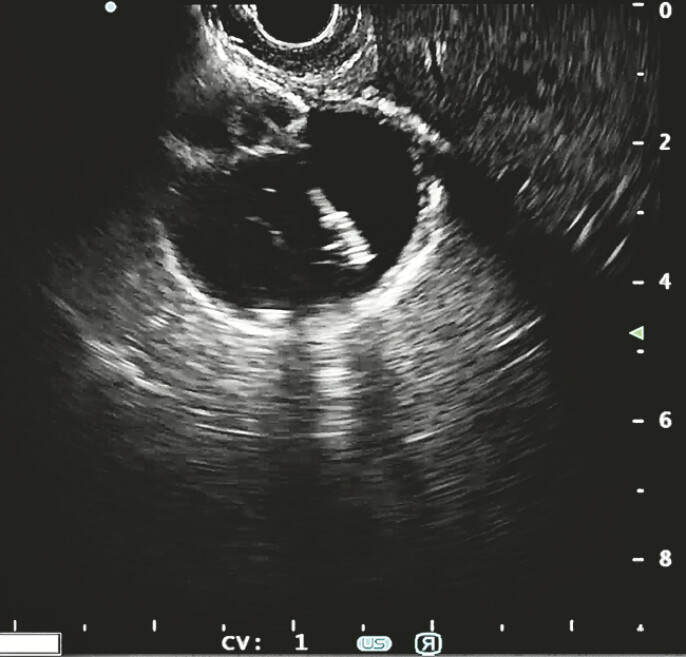
EUS-guided gallbladder drainage.

**Fig. 3:**
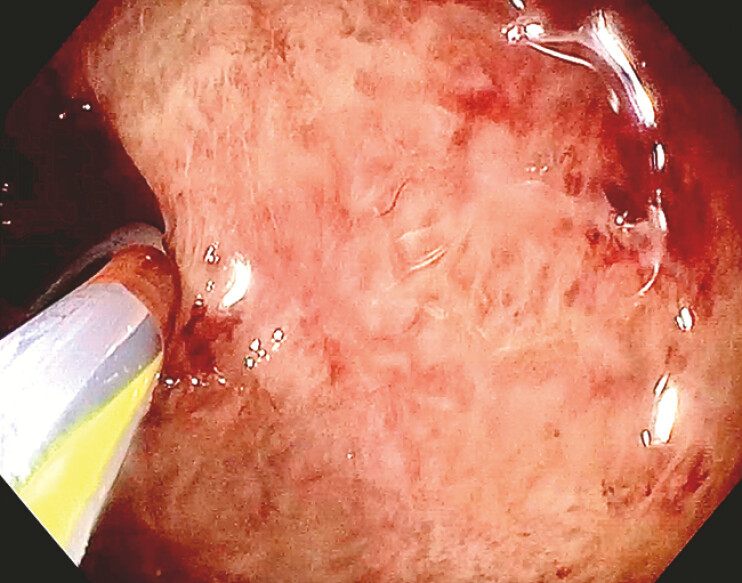
Cholecystoscopy evaluating gallbladder inflamed mucosa.

**Fig. 4:**
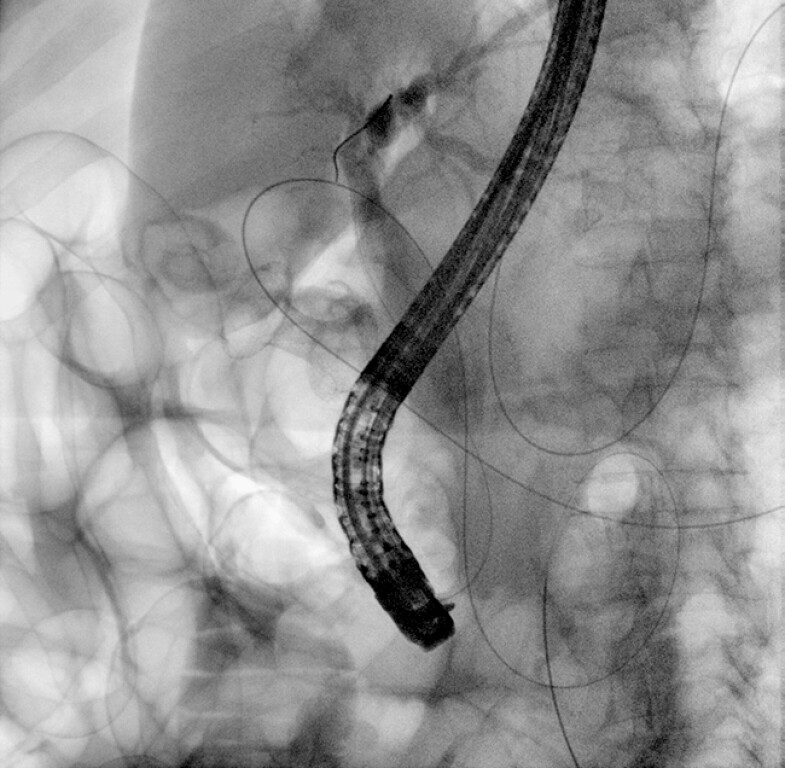
Fluoroscopic image of rendezvous from the gallbladder and retrograde biliary cannulation with a second guidewire.

This is another evidence on the potentiality of EUS-GBD with LAMS. The use of larger LAMS allowed access to the gallbladder, to perform biliary rendezvous for invisible papilla for a concomitant acute cholecystitis drainage and CBD stones clearance.

Endoscopy_UCTN_Code_TTT_1AR

## Notes

Endoscopy E-Videos https://eref.thieme.de/e-videos
*E-Videos* is an open access online section of the journal *Endoscopy* , reporting on interesting cases and new techniques in gastroenterological endoscopy. All papers include a high-quality video and are published with a Creative Commons CC-BY license. Endoscopy E-Videos qualify for HINARI discounts and waivers and eligibility is automatically checked during the submission process. We grant 100% waivers to articles whose corresponding authors are based in Group A countries and 50% waivers to those who are based in Group B countries as classified by Research4Life (see: https://www.research4life.org/access/eligibility/ ). This section has its own submission website at https://mc.manuscriptcentral.com/e-videos .
